# Uncovering the role of biogeography, resources, food web composition and time during microbial primary succession

**DOI:** 10.1098/rspb.2026.0396

**Published:** 2026-10-14

**Authors:** Benjamin Baiser, Jessica Bernardin, Erica B. Young, Grace A. Cagle, Zachary B. Freedman, Sarah M. Gray, Emmanuel Divinagracia, Isadora E. Fluck, Dominique Gravel, Shelby LeClare, Zachary T. Long, Sydne Record, Kaitlyn M. Rhyner, Leonora S. Bittleston

**Affiliations:** ^1^ Department of Wildlife Ecology and Conservation, University of Florida, Gainesville, FL 32611-0430, USA; ^2^ Biological Sciences, Boise State University, Boise, ID 83725, USA; ^3^ Biological Sciences, University of Wisconsin-Milwaukee, Milwaukee, WI 53211, USA; ^4^ Department of Soil and Environmental Sciences University of Wisconsin-Madison, Madison, WI 53706-1207, USA; ^5^ University of Fribourg, 1700 Fribourg, Switzerland; ^6^ Département de biologie, chimie et géographie, Université du Québec à Rimouski, G5L3A1 Canada; ^7^ Department of Biology and Marine Biology, University of North Carolina Wilmington, Wilmington, NC 28403-5915, USA; ^8^ Department of Wildlife, Fisheries, and Conservation Biology, University of Maine, Orono, Maine 04469, USA; ^9^ Maine Agricultural and Forest Experiment Station, University of Maine, Orono, Maine 04469, USA

**Keywords:** bacteria, food web, latent Dirichlet allocation model, microbiome, pitcher plant, primary succession, 16S rRNA, topic modelling

## Abstract

Ecological succession is shaped by multiple processes that operate simultaneously across spatial and temporal scales. While classical succession theory generally focuses on a single trophic level within a single location, often in plants or other macroscopic organisms, recent efforts have extended this framework to microorganisms across multiple ecological dimensions. The aquatic micro-ecosystem within the pitcher plant, *Sarracenia purpurea*, provides a natural model of primary succession replicated across a continental latitudinal gradient. Using this model system, we investigated how biogeography, food web structure and resource availability influenced bacterial succession. Over 12 weeks, we repeatedly sampled bacterial communities from seven sites spanning the latitudinal range of *S. purpurea* and characterized communities using 16S rRNA gene sequencing. Both taxonomic and phylogenetic diversity increased over succession. These diversity patterns were not explained by temporal changes in food web composition, resources or environmental conditions, although pH, water volume, latitude and midge abundance, each influenced diversity independently of time. Latent Dirichlet allocation models identified six compositional groups with distinct temporal and biogeographic signatures. Early-successional groups were enriched in putative diazotrophs, while late-successional groups contained metabolically diverse taxa adapted to low-oxygen conditions. Our findings show that microbial primary succession in *S. purpurea* is shaped by time, biogeography and pitcher-scale environmental filters.

## 1. Introduction

Understanding succession requires examining how multiple ecological processes operate simultaneously across spatial and temporal scales. While classical succession theory focuses on temporal changes within a single trophic level and location, successional trajectories are shaped by resource dynamics, environmental filters, trophic interactions and biogeography [1,2]. The theoretical framework for succession is based largely on early studies of plant assemblages [3–5], but has since been expanded to include other macroscopic organisms (e.g. [6]) and, more recently, microorganisms (e.g. [7–9]). For microorganisms, disentangling successional mechanisms is challenging because microbes respond rapidly to environmental change [10–12], disperse passively across large spatial scales [13] and interact with hosts and predators operating across vastly different spatial and temporal scales.

Microbial succession is influenced by biogeography through dispersal limitation and differences in regional species pools. Even with high dispersal potential, microbial communities exhibit clear biogeographic patterns across continental scales [14]. Local environmental conditions can further constrain colonization by selecting for microbial taxa with traits suited to specific physical and chemical environments, often generating phylogenetically clustered communities [15]. Food web structure and resource availability can shape succession through both top-down trophic controls and bottom-up nutrient dynamics, with food web assembly creating new niches by transforming resources and mediating species interactions [16].

As microbial succession proceeds, the relative importance of these drivers and interactions between them can shift the balance between deterministic and stochastic processes, and these differences may vary across biogeographic contexts. For example, early-successional communities may be governed by strong environmental filtering for closely related, stress-tolerant taxa or taxa with traits to deal with low nutrient conditions [17], while later succession may involve more distantly related taxa as food webs assemble and resources accumulate, creating new niches and relaxing environmental filters [16,17]. Determining the relative importance of these mechanisms at different stages of succession, and whether they vary across broad biogeographic scales, requires model systems where succession is naturally replicated over a continental range.

A compelling system for advancing theory on microbial succession in a broad ecological context is the micro-ecosystem hosted by pitchers of the carnivorous pitcher plant, *Sarracenia purpurea* [17]. The inquiline community housed in the leaves of *S. purpurea* and related subspecies is a model system in community ecology [18,19]. *Sarracenia purpurea* grows in nutrient-depleted soils and supplements mineral nutrient acquisition, especially nitrogen and phosphorus, by capturing and digesting insect prey in pitcher-shaped leaves*.* In contrast to most carnivorous plants, *S. purpurea* produces only minimal digestive enzymes and relies primarily on bacterial hydrolytic enzymes, but with support from a diverse aquatic food web composed of bacteria, fungi, protozoa, rotifers and dipteran larvae to achieve prey breakdown ([20,21]; figure 1C). Pitchers are sterile upon opening [22] and subsequently recruit their microbiomes and food webs, providing a replicated model of primary succession across the continental range of *S. purpurea,* from Florida north to Newfoundland and west to British Columbia.

**Figure 1.**
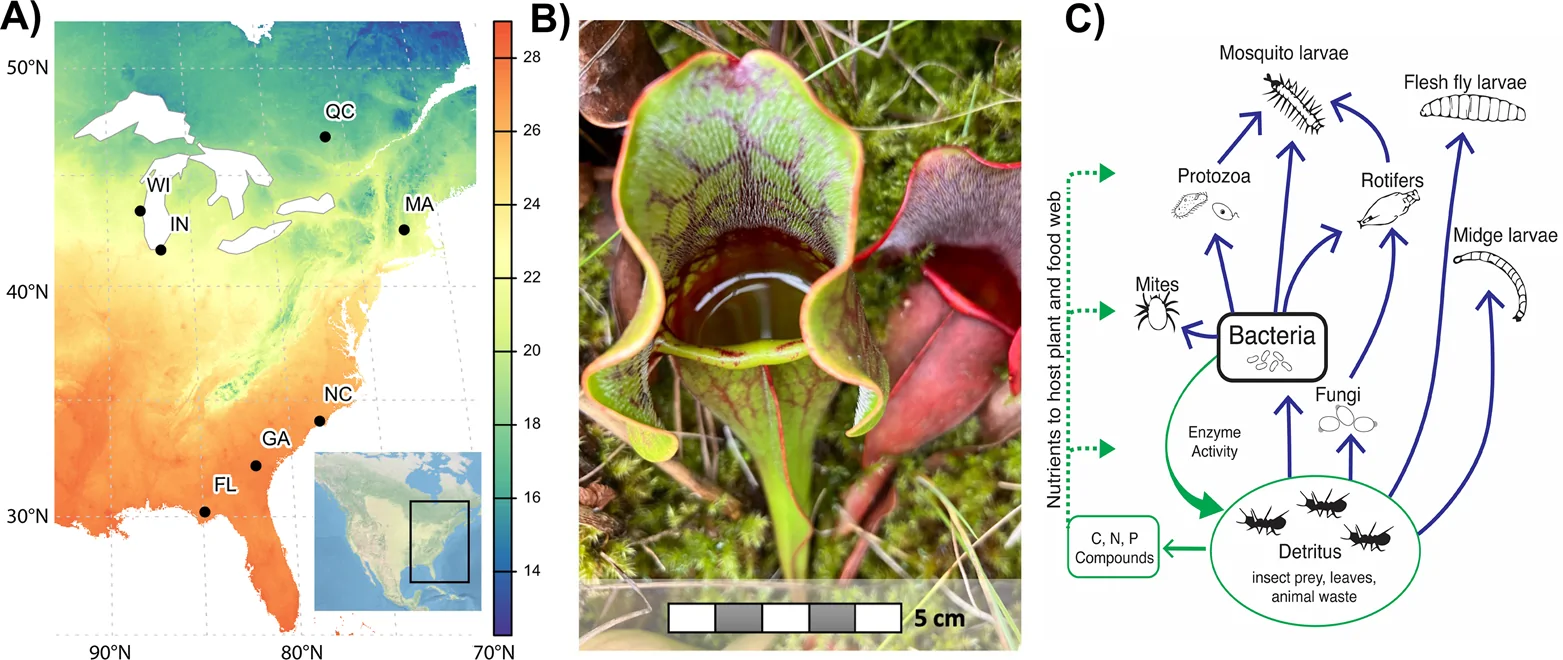
(A) Map detailing the location of the seven sampling sites: Wisconsin (WI), Indiana (IN), Quebec (QC), Massachusetts (MA), North Carolina (NC), Georgia (GA) and Florida(FL). The colour scale bar references the mean of July temperatures (°C). For more details on each site, see table S1 of appendix S1 in the supplementary material. (B) A pitcher-shaped leaf of *Sarracenia purpurea* filled with rainwater, which forms the micro-ecosystem studied. (C) Diagram of the pitcher plant food web.

We build on previous studies of succession in this system [23–26] by examining bacterial succession at higher temporal resolution and over a broader spatial range. By characterizing bacterial communities in pitchers across the plant's latitudinal biogeographic distribution, we tested how food web structure, resource availability, environmental conditions and biogeography influence bacterial succession, and whether these factors account for or interact with the effect of time. We tested the following hypotheses:

**H1** proposes that taxonomic and phylogenetic diversity change over the course of succession. H1 serves as a baseline expectation that diversity varies through time.

**H2–H4** evaluate the roles of key ecological components in the micro-ecosystem in shaping taxonomic and phylogenetic diversity during succession: **H2** focuses on the abundance of food web constituents (midge, mosquito and flesh fly larvae; figure 1C), **H3** examines the detrital resources (insect carcasses) and **H4** considers pitcher environmental conditions (pH and water volume). These hypotheses collectively test whether temporal change alone drives bacterial succession or whether time serves as a surrogate for one of the other underlying ecosystem processes. If a mechanistic temporal driver (e.g. detrital resource accumulation) is responsible, we would expect that variable to be collinear with time and for time to either drop out of the best-fit model or have reduced explanatory power.

**H5** tests statistical interactions between time and the factors in H2–H4 to determine whether the effect of time on bacterial diversity depends on food web structure, resource availability or environmental conditions. Significant interactions would indicate that succession is not governed by time alone, but by interactions with these key ecological drivers.

In **H6**, we expect early- and late-successional compositional groups (e.g. [23]) that differ in composition across the biogeographic range owing to dispersal limitation [14].

To test these hypotheses, we repeatedly sampled newly opened pitchers at seven sites (figure 1A) over 12 weeks and analysed bacterial composition using 16S rRNA gene sequencing with generalized linear mixed models (GLMMs) and latent Dirichlet allocation (LDA) models to link diversity and composition to time, biogeography, food web structure, resources and the pitcher environment.

## 2. Methods

### (a) Sites and field sampling

During summer 2022, we conducted standardized sampling of *S. purpurea* microbiome primary succession at seven sites from Florida, USA to Quebec, Canada, spanning the plant's native latitudinal range in North America (figure 1A; supplementary material, appendix S1, table S1). At each site, we identified 15 newly emerging pitchers on different plants that were about to open. On day 0, we gently ‘popped’ open each pitcher and added 7 ml sterile nuclease-free water (hereafter ‘sterile water’) to ensure that all pitchers started with similar water levels. On days 3, 7, 14, 28, 42, 56, 70 and 84 (*n* = 8 sampling periods), we resampled pitchers using a sterile transfer pipette. Sampling days varied slightly among sites owing to local conditions. From each sample, we recorded the number of intact insect prey items, mosquito (*Wyeomyia smithii*), midge (*Metriocnemius knabi*) and flesh fly (*Fletcherimyia fletcheri*) larvae, water pH (pH strip MQ Quant, Millipore Sigma) and water volume (ml). We then sub-sampled 3 ml of the pitcher water, which we stored in a cool, insulated pack for further processing. After sampling, we returned the rest of the water to the pitcher and added 3 ml of sterile water to replace the sampled volume. We centrifuged (8000 rpm, 10 min) the samples in the field or at a close-by field station soon after collection, removed all but 100 µl of the supernatant and added 300 µl RNA/DNA Shield (Zymo Research, Irvine, CA) to each sample tube.

### (b) DNA extraction, sequencing, and bioinformatics

We selected 10 pitchers with the most complete time-course of samples and metadata (i.e. some pitchers could not retain water and were excluded from further analysis) and extracted total community DNA from the water samples collected from them per site over the course of succession, using methods detailed in the supplementary materials (supplementary material, appendix S2).

We processed raw sequence data using QIIME2 v.2023.5 [27] with DADA2 [28] for quality control of sequence data. We taxonomically classified bacterial amplicon sequence variants (ASVs) of the 515f/926r region of the 16S rRNA gene using SILVA 138 [29] and produced a matrix of 484 pitcher bacterial samples with 3323 distinct ASVs. More details on bioinformatic analysis can be found in appendix S2 in the supplementary material.

### (c) Predictor variables

The predictor variables we tested were *time* since the pitcher opened in days, the number of *mosquitoes*, *midges*, and *flesh fly* larvae counted in each sample, the *latitude* and *longitude* of the sample site, the pitcher water *volume* (ml), *pH* of the pitcher fluid sampled and the number of *intact prey* items (i.e. resources in this system) counted in each sample.

### (d) α-diversity

We calculated Hill numbers to quantify taxonomic α-diversity using the *hillR* package [30]. We used the Hill number for order *q* = 0 (i.e. richness) for analyses and results in the main text because orders *q* = 0, 1 and 2 were highly correlated (results for *q* = 1 and *q* = 2 are in tables S3 and S4 of appendix S3 in the supplementary material). We quantified phylogenetic α-diversity as the standard effect size of Faith's phylogenetic diversity (SES PD) using the function ‘ses.pd’ with the ‘tip-swapping’ method in the R package *picante* [31]. We used the ASV pool for each site to calculate SES PD for the pitchers in the given site.

We analysed how ASV richness and SES PD were influenced by time, biogeography, resources and food web composition along a successional trajectory using GLMMs with the function ‘glmmTMB’ in the R package *glmmTMB* (v.1.1.9; [32]). The full model for ASV richness included *time*, a quadratic term for *time*, interactions between *time* and *pitcher volume*, *time* and *pH*, *time* and *intact prey*and *time* and *midge*, *mosquito* and *flesh fly* larval abundances. The quadratic term accounts for potential mid-successional peaks in prey capture of first-year leaves [33]. We also included longitude and latitude as fixed effects and *pitcher Id* nested in *site* as random intercepts. Predictor variables were scaled to a mean = 0 and s.d. = 1. The richness GLMM had a Gaussian error distribution and identity link function. We checked model assumptions using the *DHARMa* package (v.0.4.6; [34]) and identified that the residuals of the full model were heteroscedastic. We modelled dispersion for *time*, *latitude* and *longitude* to account for their changing dispersion in the full model. We then performed variable selection with a likelihood ratio test using the ‘drop1’ function in the *stats* package [35] to select the most parsimonious model. We also calculated variance inflation factors (VIF) using the ‘check_collinearity’ function in the *performance* package in R (v.0.12.4; [36]) to determine whether time was collinear with the other predictor variables. We used the same model building procedure for the SES PD model, the only difference being that the dispersion model only included *longitude*.

### (e) Latent Dirichlet allocation model to identify compositional groups

We investigated successional changes in the composition of pitcher plant microbiomes using the LDA model [37] to identify groups of bacterial taxa and relate these groups to our predictor variables. The LDA model was originally devised for text mining with the goal of determining the latent *topics* of a *document* based on the frequency of *words* that appear together. Valle and colleagues [38] adapted this approach to biodiversity data to characterize a sampling unit (document) in terms of its latent compositional groups (topics), where each latent group (topic) corresponds to a distribution of species (words). The LDA model is a Bayesian mixed-membership model that differs from standard multivariate methods for clustering (e.g. *k*-means clustering, hierarchical clustering) or testing groups (e.g. PERMANOVA) by allowing a sample (pitcher at a time point in this case) to be assigned to more than one cluster/group. Because the LDA model allows samples to have varying contributions from multiple LDA groups, these groups should be interpreted as dominant or recurring compositional groupings rather than distinct or discrete communities.

We conducted LDA on the pitcher-by-ASV matrix using the *ldacov* package [39]. We used the truncated stick-breaking prior to determine the optimal number of groups [40] using the ‘gibbs.lda’ function in the *ldacov* package. For the optimal number of groups, we then ran the ldacov model with the ‘gibbs.lda.cov’ function. The ldacov model has three important outputs. The first is a set of posterior distributions for the regression coefficients that relate each covariate in the model to each latent group. The second output is the *Φ* matrix, which contains the distributions of the ASVs within each latent group (e.g. supplementary material, appendix S4, figure S1). The third output is the *θ* matrix, which contains the distributions of each latent group in each sampling unit (i.e. pitcher sample, e.g. supplementary material, appendix S4, figure S2). With these three pieces of information, we can infer which predictor variables are most important for each latent group, which ASVs are most abundant in each latent group and how latent group distributions change across samples.

### (f) Latent group membership

To explore the LDA groups, we tested them for phylogenetic signal with Pagel's lambda [41] and Blomberg's *K* [42] and used non-metric multidimensional scaling (NMDS) on a Wisconsin-transformed Bray–Curtis distance matrix to visualize LDA group composition. Testing for phylogenetic signal was performed under three filtering scenarios, in which taxa were included only if they exceeded a probability threshold of 0.01, 0.001 or 0.0001 (i.e.* Φ* > 0.01, *Φ* > 0.001 and *Φ* > 0.0001) of belonging to at least one LDA group. We built a single phylogenetic tree containing all ASVs using SEPP for multiple-sequence alignment and FastTree for phylogenetic reconstruction within QIIME2, and for each probability threshold, we filtered the tree to include only ASVs exceeding that threshold [43,44].

To gain insight into potential life history functions of the LDA groups, we examined the taxa identified from the 10 most abundant ASVs in each LDA group using SILVA v.138 taxonomic classifications. We used BLAST to manually confirm and update taxonomy [45] and conducted a database and literature search to ascribe species, genus or family of taxa to associated functions, habitats and abiotic tolerances. We further used the functional annotation of prokaryotic taxa (FAPROTAX) program [46] to identify some potential habitat preferences and predicted functions associated with these dominant ASVs.

## 3. Results

### (a) Modelling α-diversity

The best-fit model for richness included *time*, the quadratic for *time, volume, pH, latitude, longitude, intact prey,* an *interaction between time* and *intact prey* in the conditional model and *time, latitude and longitude* in the dispersion model (supplementary material, appendix S3, table S1). Bacterial richness showed a significant quadratic relationship with *time* (time, *z* = 22.85, *p* < 0.0001; time^2^, *z* = –4.34, *p* < 0.001), increasing over the course of succession (figure 2A). Bacterial richness increased with *pH* (*z* = 3.34, *p* < 0.001; figure 2B) and decreased with *volume* (*z* = –2.65, *p* < 0.001; figure 2C). Bacterial assemblages had greater richness at more westerly longitudes (*z* = –3.76, *p* < 0.001; figure 2E). Bacterial richness showed a marginal increase with *latitude* (*z* = 1.83, *p* = 0.07; figure 2D) and a marginal decrease with the interaction between *time* and *intact prey* (*z* = –1.69, *p* = 0.09; figure 2A). The dispersion model (supplementary material, appendix S3, table S1) showed that variance in bacterial richness significantly increased with *time* (*z* = 7.36, *p* < 0.0001) and *latitude* (*z* = 4.13, *p* <0.001) and decreased with *longitude* (*z* = –9.17, *p* < 0.0001). Variance inflation factors were low (<2), indicating that multi-collinearity was not an issue and that resources, food web structure and pitcher environment were not collinear with time.

**Figure 2.**
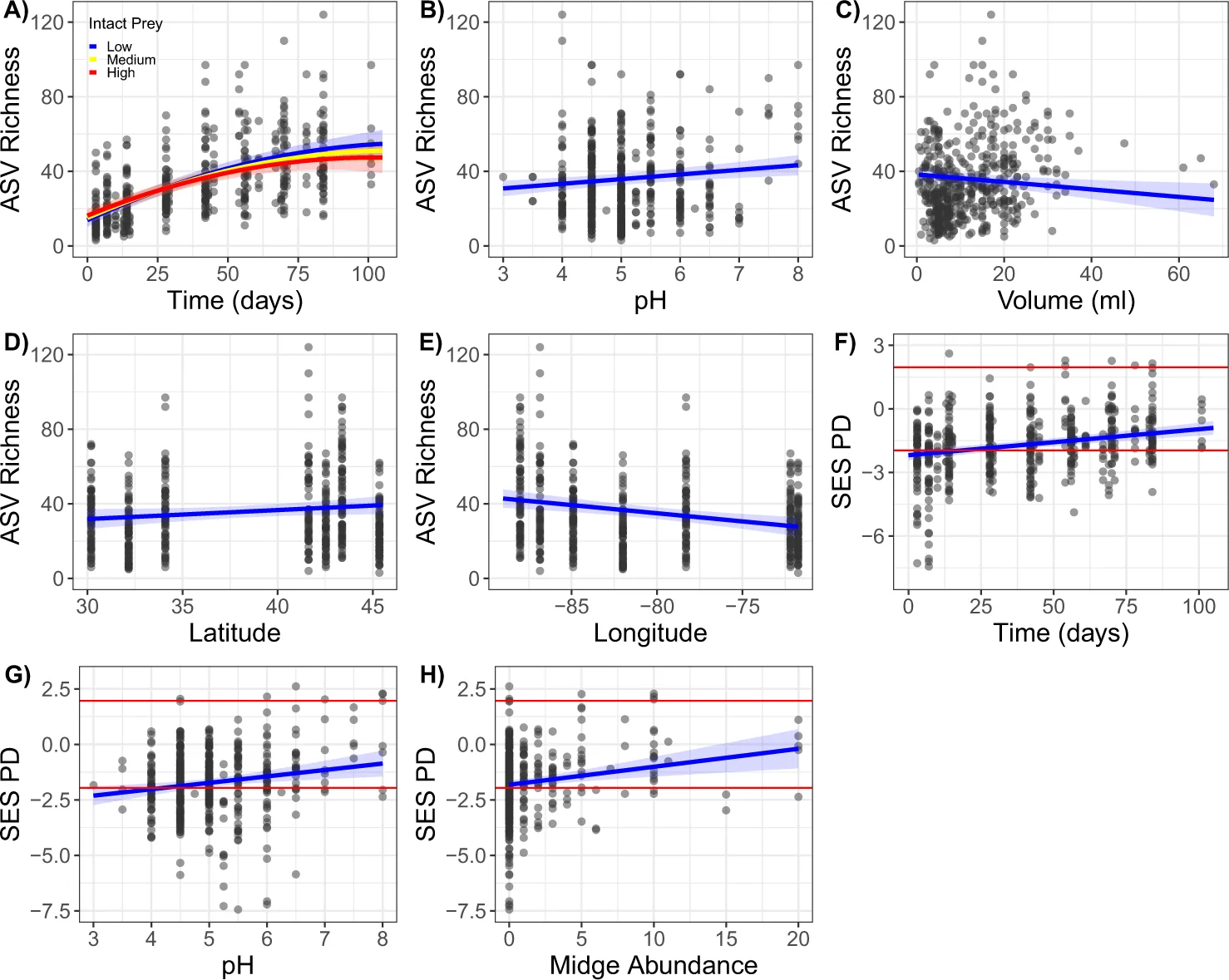
Change in richness and phylogenetic diversity during succession. GLMM predictions showing the relationships between ASV richness and (A) time, (B) pH, (C) volume, (D) latitude, (E) longitude and SES PD, (F) time, (G) pH and (H) midge abundance. All other variables in the models (supplementary material, appendix S3, tables S1 and S2) were held constant at their mean for these predictions. Raw data points are shown in grey and ribbons indicate 95% CIs. Horizontal red lines on (F), (G) and (H) indicate SES PD values of ±1.96.

The best-fit model for the standardized effect size of phylogenetic diversity (SES PD) included *time, pH, midge* abundance and *longitude* (supplementary material, appendix S3, table S2). VIF values were also low (<2) in the SES PD model. Bacterial SES PD showed a significant increase with *time* indicating an increase in phylogenetic diversity over succession (*z* = 6.18, *p* < 0.001; figure 2F). While nearly all bacterial assemblages were phylogenetically under-dispersed (i.e. SES PD values < 0), early-successional assemblages tended to exhibit significant phylogenetic under-dispersion (i.e. SES PD values < –1.96) more frequently than later-successional assemblages (figure 2F). SES PD had a positive relationship with *pH* (*z* = 3.06, *p* = 0.002; figure 2G) and *midge* abundance (*z* = 3.47, *p* < 0.001; figure 2H). The dispersion model indicated that variance in bacterial SES PD significantly decreased with *longitude* (*z* = –4.00, *p* < 0.001). Observed successional trajectories for each component of the ecosystem are shown in figure S1 and S2 of appendix S5 in the supplementary material.

To assess bacterial succession in the pitcher plant microbiome at a greater taxonomic scale, we plotted the mean relative abundance of the 10 most abundant bacterial taxonomic classes across all 70 pitchers at each time point during succession (figure 3). The classes Alphaproteobacteria, Bacteroidia and Gammaproteobacteria consistently had the highest relative abundances across the successional trajectory. Gammaproteobacteria and Bacteroidia peaked early and declined, whereas Clostridia and other classes increased later in succession (figure 3).

**Figure 3.**
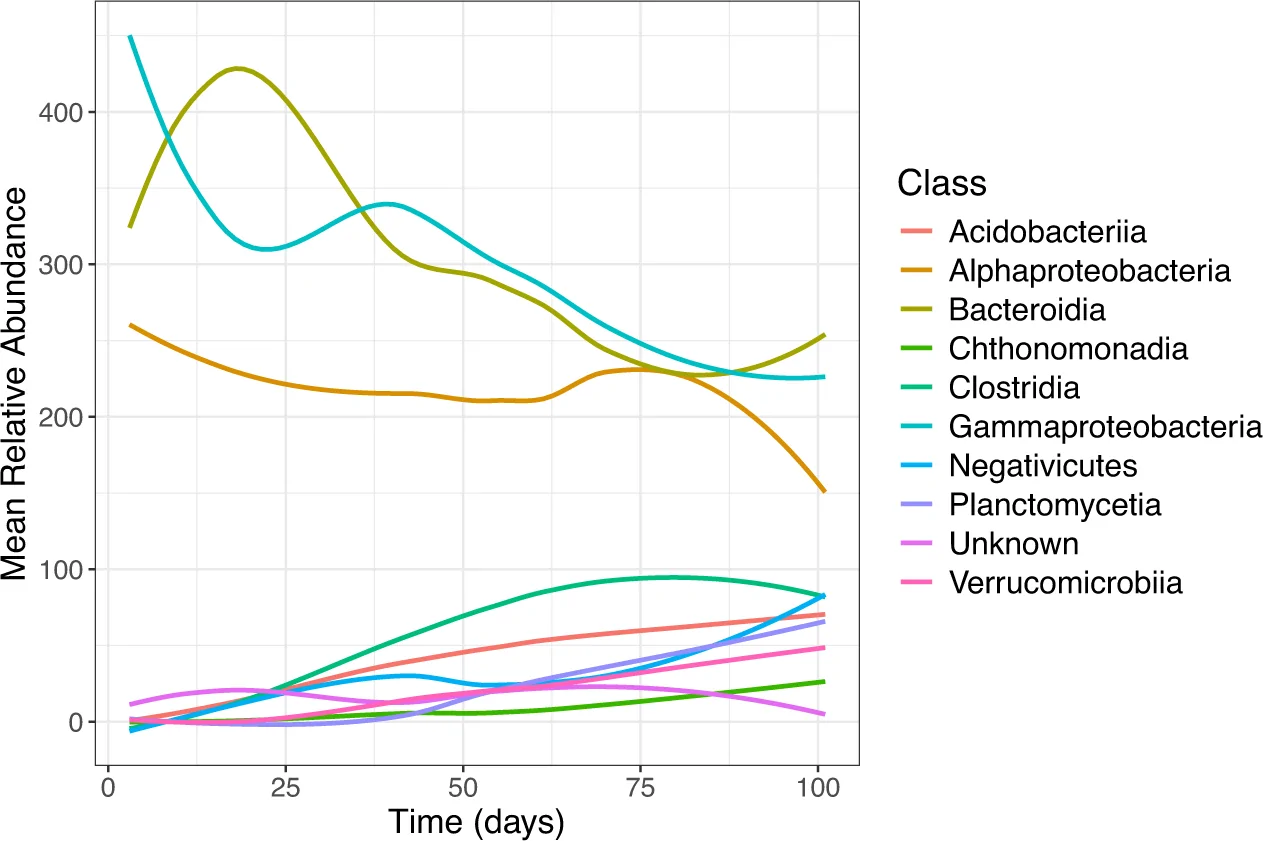
Mean relative abundance for the 10 most abundant bacterial classes across the successional trajectory of the *S. purpurea* microbiome. Lines are fit using locally estimated scatterplot smoothing (LOESS).

#### (i) LDA modelling of composition

Using the truncated stick-breaking prior with a maximum of 10 groups, we identified six groups as the optimal group number for the LDAcov model. These six groups contained over 75% of the elements in the pitcher-by-ASV matrix. Taxa with high probabilities of belonging to LDA groups tended to be concentrated in the Gammaproteobacteria, Bacteroidia and Alphaproteobacteria classes (supplementary material, appendix S6, figure S1). These were the top three classes for all LDA groups except group 4, which was dominated by taxa belonging to Bacteroidia, Gammaproteobacteria and Clostridia.

While we found no phylogenetic signal within our LDA groups using Blomberg's *K* (supplementary material, appendix S6, table S1), Pagel's lambda values varied substantially among groups (supplementary material, appendix S6, table S2). Most LDA groups had lambda values very close to zero (*λ* = 0.01–0.06; supplementary material, appendix S6, table S2). Although several of these groups had significant adjusted *p*-values (*p* < 0.05), the small lambda values indicate that any detected phylogenetic signal was weak and may be driven by sample size rather than meaningful phylogenetic structure. By contrast, LDA group 3 showed a moderate phylogenetic signal, with lambda values ranging from 0.64 to 0.22, and the signal was significant (adjusted *p* < 0.05) across all three probability thresholds (supplementary material, appendix S6, table S2).

The LDA model allowed us to link covariates to the probability that a group will be present in a pitcher. Overall, *time*, *latitude* and *pH* were the most important predictor variables for our latent bacterial groups (figure 4). There were early- and late-successional groups at both high and low latitudes and groups that favoured both lower and higher pH environments (pitcher water pH ranged from 3 to 8). LDA group 1 was a low-latitude, late-successional group (figure 4A) present at low pH. Group 2 was found predominantly in high-latitude pitchers with low pH and fewer midges, but did not show a significant temporal relationship (figure 4B). LDA group 3 was an early-successional, low-latitude group that favoured low pH and tended to occur in more easterly sites (figure 4C). Group 4 was late-successional, high-latitude and occurred more often at higher pH (figure 4D). LDA group 5 was an early-successional, high-latitude group that preferred low-volume pitchers with higher pH and occurred more frequently at westerly sites (figure 4E). Finally, LDA group 6 was an early-successional, high-latitude group (figure 4F) that occurred more often at easterly sites and was associated with pitchers containing fewer mosquito larvae and fewer intact prey items.

**Figure 4.**
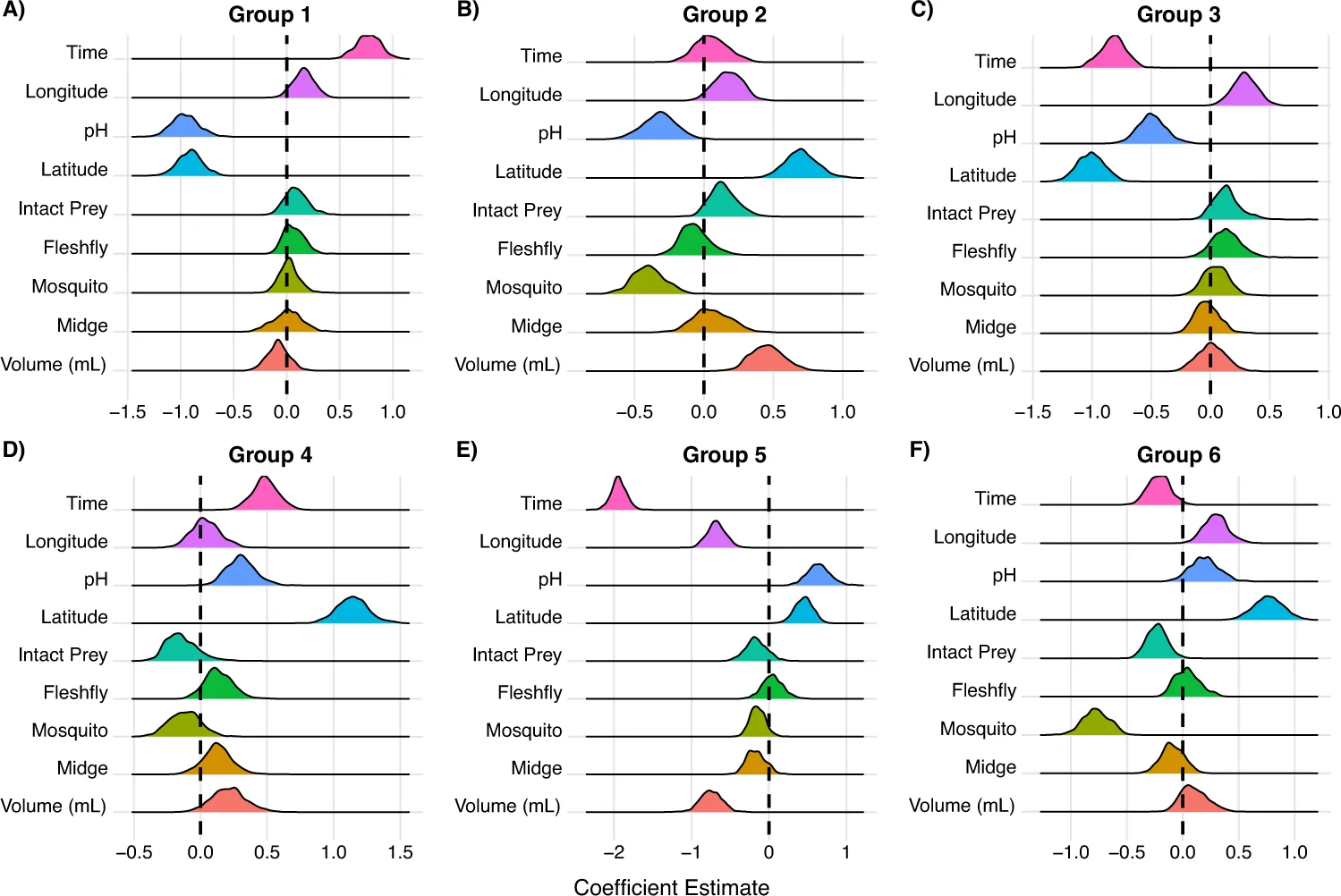
Posterior distributions of coefficient estimates from the LDAcov model detailing the relationships between each LDA group (A–F) and the predictor variables. Distributions whose 95% credible intervals do not overlap zero are considered significant. Mean values for each distribution arein table S1 of appendix S4 in the supplementary material.

The NMDS plot of LDA groups (supplementary material, appendix S7, figure S1) reinforces the temporal and latitudinal group associations from the LDA model. The first NMDS axis ordinated LDA groups based on latitude, with high-latitude groups 6, 2 and 4 having positive scores and low-latitude groups 1 and 3 having negative scores (supplementary material, appendix S7, figure S1). The second NMDS axis arranged sites based on their successional trends. Late-successional LDA groups 1 and 4 had negative scores while early-successional groups 3, 5 and 6 had positive scores on the second NMDS axis (supplementary material, appendix S7, figure S1).

To gain further insight into composition and function, we explored the taxonomic identities and functions associated with the ten most abundant ASVs in each LDA group (supplementary material, appendix S8, table S1). LDA group 1 (late-successional) included obligate and facultatively aerobic bacteria *Mucilaginibacter*, *Dinghuibacter, Paludibacterium* and *Puia,* which can degrade polysaccharides, organic phosphate and other compounds. Group 1 also notably included the obligate anaerobe *Clostridium* and microaerophilic *Telmatospirillum,* which may support prey hydrolysis under low-oxygen conditions that occur with putrefaction. LDA group 2 (no relationship with time) included members of *Rhodanobacter*, *Novosphingobium* and *Chromobacterium*, which have also been associated with pitcher-plant mosquito larvae [23,47]. Group 2 also included *Dyella* and *Rhodanobacter*, which are linked to sulphur oxidation, along with *Mucilaginibacter*, *Chryseobacterium* and *Chromobacterium*, which are associated with nitrate reduction. LDA group 3 (early-successional) included mostly aerobic or facultatively aerobic taxa, including *Mucilaginibacter* and *Chryseobacterium* with capacity for nitrate reduction and chitinolytic potential and *Massilia* with capacity for cellulose degradation and N_2_ fixation. LDA group 4 (late-successional) included an obligate anaerobe (*Clostridium*) and taxa that can use complex organic compounds (e.g. *Chitinophagaceae*), including those produced in putrefaction. This group included the acidophilic Sphagnum bog bacteria, *Schlesneria,* which can also degrade diverse polymers [48]. Abundant taxa in group 5 (early-successional) included those that can use diverse carbon sources (e.g. *Sphingomonas and Mucilaginibacter*), plant-associated taxa that potentially fix N_2_ (*Rhizobium*, *Janthinobacterium* and *Burkholderia* clades). Common taxa in group 6 (early-successional) included insect-associated taxa (e.g. *Flavobacterium cerium*) and a genus specifically associated with pitcher plant mosquito larvae (*Elizabethkingia*; [47]), along with taxa that show nitrate reduction potential, including *Pseudomonas, Hymenobacter* and four *Flavobacterium* ASVs.

Importantly, the latent bacterial groups derived from the LDA model can co-occur simultaneously within pitchers, allowing for a more nuanced interpretation of primary succession. To illustrate this, we focused on the successional trajectories of LDA groups within individual pitchers that exemplified the most abundant LDA groups at different time points across each of our seven sites (figure 5). Information on what proportion of the entire community each LDA group comprises is taken from the *θ* matrix [38]; supplementary material, appendix S4, figure S2). Focusing on the Florida pitcher (figure 5), succession began with group 5 making up >95% of the community and group 3 present at a low proportion. By day 7, group 5 drastically declined and group 3 comprised approximately 60% of the community, with LDA groups 1, 2 and 5 each making up between 10–15%. From sampling day 28 onwards, LDA group 1 dominated, with proportions exceeding 70% and approaching 100% by the end of the successional trajectory (figure 5). Groups 2 and 3 were present at lower proportions until sampling day 56 (figure 5). Based on the dominant LDA groups across this pitcher's trajectory, we characterize this successional trajectory as 5 to 3 to 1 (figure 5). This was the most common successional trajectory at the Florida site.

**Figure 5.**
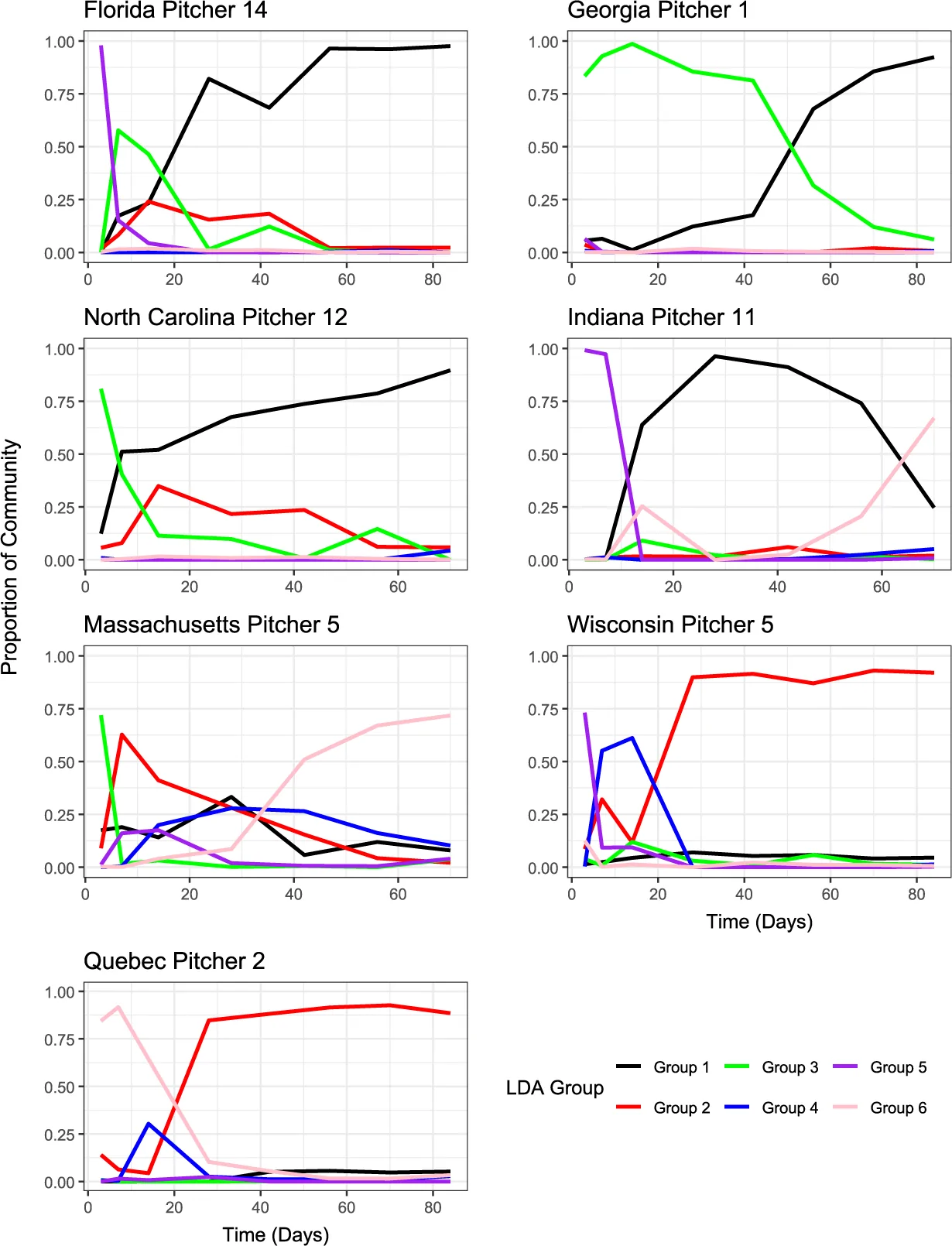
Individual pitchers showing the most common successional trajectories of the six latent groups identified by the LDA model within each site.

Across all pitchers, the most frequent trajectory was dominance by LDA group 3 followed by group 1, which occurred in 19% of pitchers and was concentrated in the south-eastern sites of Georgia and North Carolina (figure 5). The successional trajectories 5 to 3 to 1 and 6 to 2 (the most prevalent trajectory at the Quebec site; figure 5) were the next most frequent, each occurring in 7% of pitchers. Overall, LDA groups 3, 5 and 6 dominated early succession, representing the highest-proportion group in initial samples in 39%, 23% and 23% of pitchers, respectively. LDA group 1 most frequently emerged as the dominant group by the final time point, dominating the end of the successional trajectories in 49% of pitchers across all sites. However, LDA group 1 did not occur in Wisconsin or Quebec, where group 2 was most commonly dominant at the end of succession (figure 5).

## 4. Discussion

### (a) α-diversity

We found that ASV richness increased with time, supporting H1 that diversity changes over the course of succession. A positive richness–time relationship is expected as sterile pitchers are colonized and begin capturing prey. The system experiences exogenous, heterotrophic succession [8] where resource (i.e. invertebrate prey) capture peaks between 12–33 days [33]. The quadratic term for time indicates that the rate of increase in taxonomic richness slows over time, suggesting that the richness–time relationship may eventually asymptote or turn negative (i.e. a hump-shaped richness–time relationship). While our approximately 84-day sampling period represents part of a pitcher's 2-year lifespan [33], it captured the transition through peak and decline of prey capture in first-year pitchers [25,33]. An increasing relationship that asymptotes and then decreases much later in succession was also observed for bacterial α-diversity in the pitcher plant *Darlingtonia californica* [49].

To gain a mechanistic understanding of the time–richness relationship, we assessed the temporal dynamics of the food web, resources and environmental conditions in the *S. purpurea* micro-ecosystem. We found little evidence that change in measured ecosystem components over time drove ASV richness. Time remained the strongest predictor in the best-fit model and showed low collinearity with other variables, indicating that it was not acting as a surrogate for the factors we measured. Further, there were no significant interactions between time and food web components, resources or environmental conditions, providing no support for H5.

The environmental components of the ecosystem (i.e. pH and volume) influenced ASV richness, although these effects did not follow a temporal trend. Greater pitcher water volume was associated with lower bacterial richness, whereas higher pH resulted in greater ASV richness. The decrease in bacterial richness with greater pitcher volume may be attributable to several ecological mechanisms. As pitcher microbial communities are mainly fuelled by prey inputs, larger water volumes can dilute dissolved nutrients, which can lead to relatively reduced bacterial richness [50,51]. Further, greater water volume may serve to reduce environmental fluctuations, resulting in relatively reduced niche availability, which could in turn support lower bacterial richness [52]. Pitcher water tended to be acidic, with an average pH of approximately 5 (range 3–8). The increase in ASV richness with pH was probably owing to acid stress driving the need for physiological pH management, including proton pumps, thus limiting the number of taxa surviving at the lower pH levels [53].

In contrast with previous studies in the pitcher plant system [20,23,54,55], we found no significant direct influence of midge or mosquito abundance on ASV richness. ASV richness increased with latitude, which contrasts with the richness–latitude relationship for pitcher plant microbes found by [54] and most other taxa [56], but supports the findings of [57] for pitcher plant microbes identified by morphotype. We also observed that richness increased at more westerly longitudes, which may reflect differences in regional species pools, dispersal limitation or unmeasured environmental gradients that co-vary with longitude across the range of *S. purpurea*.

Phylogenetic α-diversity also increased over the course of succession, providing support for H1. Similar to the richness results, time was not a surrogate for any of the other ecosystem components as it remained in the best-fit model and was the most influential variable. While most pitchers had lower than expected SES PD across the successional trajectory, early-successional samples (i.e. days 3 and 7) showed more instances of significant phylogenetic under-dispersion and had the largest magnitude of phylogenetic under-dispersion. This result suggests that early-successional, low-nutrient conditions filter for closely related taxa, probably in the classes Actinobacteria, Bacteroidia and Gammaproteobacteria. While prey is captured and the food web assembles, these filters are relaxed, allowing for more distantly related taxa to colonize and co-exist, potentially owing to the creation of new niches. Midge larvae, which had a positive effect on SES PD, create new niches by shredding prey via their role in the ‘processing chain commensalism’ [58]. However, the mechanism by which niche diversity increases with succession and leads to greater SES PD assumes that distantly related microbial taxa possess different traits that allow them to exploit newly available niches. Further, SES PD increased with pH, probably because a greater diversity of taxa can thrive at the more neutral pH levels of approximately 6–8, while acidophilic bacteria in the *S. purpurea* bacterial community are restricted to fewer phylogenetic groups.

The temporal relationships that we observed in bacterial classes reflect the increase in phylogenetic α-diversity during succession, with less dominance by the three most abundant classes (Gammaproteobacteria, Bacteroidia and Alphaproteobacteria) later in succession. Decreases in relative abundances of Gammaproteobacteria during succession have also been documented in a short three-month successional period on cottonwood leaves [59], and in three long-term chronosequences: in a salt marsh [60], during the conversion of river tidal wetlands to rice paddies [61] and in post-glacial succession [62]. Gammaproteobacteria might display early dominance because it is the most physiologically diverse class of the Pseudomonadota, as well as the most genus-rich class of bacteria; thus, it could be present in greater numbers just by chance or owing to the group's high niche diversity. In our study, the most abundant taxa in the class Gammaproteobacteria in early succession were in the order Burkholderiales. This order contains freshwater-associated and nitrogen-fixing taxa that may do well in the early-successional, lower-nutrient pitcher water. The class with the clearest increase in mean relative abundance over time was Clostridia, which primarily includes obligate anaerobes. Anaerobic conditions that are necessary for Clostridia taxa to thrive increase over time because prey capture, bacterial abundance and decomposition increase oxygen demand in the pitcher plant micro-ecosystem [63]. Within class Clostridia, the obligate anaerobe *Clostridium* was identified in late-successional assemblages. Other systems with decreasing oxygen over time, such as carcass decomposition [64,65] and the conversion of tidal wetlands to rice paddies [61], also observed increases in Clostridia in later successional stages.

The increase in bacterial taxonomic and phylogenetic diversity over time has several possible explanations, although we did not identify an underlying temporal driver among the variables we measured. First, it is possible that we failed to measure an important temporal driver of phylogenetic and taxonomic diversity in our study for which time is acting as a surrogate. One possibility is that the availability of nutrients, such as nitrogen, phosphorus or carbon, is a more informative predictor of bacterial diversity than intact prey alone. Intact prey reflects only the amount of unprocessed resource, whereas direct measurements of dissolved nutrients can capture both the quantity of prey and the processing of prey by the food web [21,66]. Because nutrient concentrations integrate decomposition, microbial activity and mineralization [67], they may show a clearer temporal signal that aligns with changes in phylogenetic and taxonomic richness. Another possibility is that successional dynamics do not follow a simple linear timeline because the amount and quality of resources available to bacteria depend not only on prey capture but also on the assembly of a food web that processes those resources [21,58]. Following prey capture, *M. knabi* shreds prey carcasses, increasing substrate availability for bacteria, which in turn support higher trophic levels, including *W. smithii*. The food web also contributes nutrients to both the plant and microbial community through nitrogen-rich waste [23,68]. Taken together, this complexity suggests that bacterial succession unfolds through overlapping and asynchronous processes that generally increase taxonomic and phylogenetic diversity and produce several common compositional trajectories (e.g. LDA groups 5 to 3 to 1; 3 to 1; 6 to 2) without revealing a single temporal driver beyond time itself.

### (b) LDA

Our LDA approach to characterizing the composition of the *S. purpurea* microbiome revealed six latent groups. These groups provide insight into both the succession and biogeography of the *S. purpurea* microbiome. Groups 1 and 4 were late-successional, and groups 3, 5 and 6 were early-successional, indicating clear shifts in microbiomes during succession. In addition to time, biogeography influenced compositional groups supporting H6 that successional groups differ in composition across the biogeographic range. There were both early- and late-successional groups that were affiliated with high and low latitudes. While the macrobial food web is found across the entire range of the plant [14,69,70], our results indicate that there is clear dispersal limitation for the microbial taxa hosted by *S. purpurea*. Our latent groups suggest dispersal limitation at greater distances, but simultaneously we found evidence of strong selection for early- and late-successional groups at both high- and low-latitude sites.

Local conditions measured within each pitcher also influenced LDA groups. pH correlated with five of the six groups, with low- and high-pH groups across nearly all combinations of early–late- successional and high–low latitude groups. The importance of pH is unsurprising, as numerous studies have observed strong effects of pH on bacterial community composition, from soil systems to pitcher plant micro-ecosystems [71,72]. *Sarracenia purpurea* pitcher water is usually mildly acidic, probably owing to the acidity of the surrounding bog, but it can shift with prey capture (e.g. ant prey can decrease the pH) or microbial activity, so the directions of pH shifts are not always clear. In our results, groups 2 and 3 were both correlated with lower pH and contained taxa from acid-tolerant or acidophilic groups (e.g. *Mucilaginibacter, Chryseobacterium* and *Rhodanobacter*) in their 10 most abundant ASVs. Of the macrobial species in the food web that we quantified, only mosquito larval abundance influenced LDA groups, having a negative effect on groups 2 and 6. The presence of mosquito larvae is known to decrease taxonomic and phylogenetic α-diversity [23,55], but results in more heterogeneous microbial communities (i.e. greater β-diversity across pitchers) in the *S. purpurea* micro-ecosystem [55].

The most abundant taxa in each LDA group allowed us to generate insights into functional dynamics during succession. All groups included taxa with strong hydrolytic enzyme potential, which is a core ecosystem function of bacterial communities that break down and metabolize cellulose, chitin, lignin and other complex biomolecules [21]. This activity is consistent with detrital decomposition releasing nutrients that sustain the food web and the host plant in *S. purpurea* pitchers [73]. Nevertheless, our results suggested that there may be differences in functional potential among LDA groups. Early-successional groups included putative diazotrophs while late-successional groups were often more metabolically diverse, with taxa capable of photoheterotrophy and chemoheterotrophy, and taxa tolerant of the low-oxygen conditions arising during prey decomposition. However, phylogenetic conservation of microbial functional traits can be variable, with some traits like N_2_ fixation being relatively highly phylogenetically conserved and other traits like those associated with C substrate usage being less conserved [74]. Therefore, functional trait estimations based on taxonomic data are limited, and more direct measurement of functional process rates or gene expression profiles (e.g. enzyme activity assays, metagenomics and metatranscriptomics [66,75]) would serve to further enhance understanding of microbial functional primary succession.

Our results support prior observations that distinct mechanisms govern early- versus late-successional communities of bacteria. In other microbial systems, there is evidence of both the dominance of stochastic [76] and deterministic [77] processes in early succession; which one dominates is probably dependent on the extent of selection in that particular environment. A recent cross-system meta-analysis of microbial primary succession determined that as succession proceeds, a greater degree of environmental filtering related to habitat-specific conditions often occurs [78]. In *S. purpurea* pitchers, the host pitcher tissue probably serves as an active environmental filter [66,79] that drives environmental selection along with low-nutrient conditions in newly opened pitchers. In our study, some early-successional LDA groups were associated with low pH conditions, while others were associated with higher pH, indicating that these assemblages are not simply stochastic subsets of the regional species pool, but instead reflect habitat-specific environmental filtering [78,80].

Similarly, some late-successional LDA groups were associated with low pH conditions, while others were associated with higher pH, supporting the idea that these communities are shaped by deterministic, habitat-specific environmental filtering [78]. In late succession, competition probably acts more strongly, and facilitation may also become more important [77,78], which could lead to a greater degree of phylogenetic overdispersion or a decrease in phylogenetic clustering, as we observed in our study. However, this assumes that competition is stronger between more closely related organisms and that facilitation is more likely to occur between more distantly related bacteria, which have been observed in microbial communities (e.g. [81]).

*Sarracenia purpurea* provides an opportunity to study microbial primary succession across replicate micro-ecosystems at a continental scale. We found that taxonomic and phylogenetic diversity increased over the course of succession. Although we did not identify a temporal mechanism underlying the diversity patterns, future work that directly measures dissolved nutrients in the pitcher fluid may better capture the ecological processes occurring in this micro-ecosystem and reveal a mechanistic temporal driver of bacterial succession. Biogeography and time also influenced microbial composition during succession, with early- and late-successional groups at both high- and low-latitude sites. The novel application of LDA to microbiome succession provided important insights into how early- and late-successional groups can co-occur throughout succession, with certain groups of bacterial taxa dominating at different, somewhat predictable times. A next step in this research would be to more fully characterize functional processes during succession beyond taxonomic composition (e.g. using metagenomics and metatranscriptomics) to better understand the impacts of successional changes on the microbial community, the food web and the host plant. Ideally, a functional approach will allow for broader generalizations about microbiome succession in the pitcher plant system and more generally in the succession of microbiomes across diverse ecosystems.

## Supplementary Material



## Data Availability

Data and code are available at Zenodo [82]. Raw 16S rRNA sequencing reads are available through NCBI BioProject PRJNA1164312, Primary succession of the Sarracenia purpurea microbiome across its continental range. Supplementary material is available online [83].
