# Supplementary material for: Uncovering the role of biogeography, resources, food web composition and time during microbial primary succession
Source: Proc Biol Sci. 2026 Oct 14;293(2080):20260396. doi: 10.1098/rspb.2026.0396 (PMC13678520; doi:10.1098/rspb.2026.0396)
Supplement: Supplementary file 1 [file rspb.2026.0396.zip › rspb.2026.0396/rspb20260396_si_001.docx]

**Supplemental Materials**

**Journal: Proceedings of the Royal Society B**

**Submission Type: Research Article**

**Title:** Uncovering the role of biogeography, resources, food web composition, and time during microbial primary succession

https://doi.org/10.1098/rspb.xxxx

Benjamin Baiser^*1^, Jessica R. Bernardin^2^, Erica B. Young^3^, Grace A. Cagle^4^, Zachary B. Freedman^4^, Sarah M. Gray^5^, Emmanuel Divinagracia^3^, Isadora E. Fluck^1,6^, Dominique Gravel^7^, Shelby LeClare^1^, Zachary T. Long^8^, Sydne Record^9^, Kaitlyn M. Rhyner^3^, Leonora S. Bittleston^2^

**Affiliations**:

^1^Department of Wildlife Ecology and Conservation, University of Florida, Gainesville, Florida, USA

^2^Department of Biological Sciences, Boise State University, Boise, ID USA

^3^Department of Biological Sciences and School of Freshwater Sciences, University of Wisconsin-Milwaukee, Milwaukee, Wisconsin, USA.

^4^Department of Soil and Environmental Science, University of Wisconsin-Madison, Madison, Wisconsin, USA

^5^Department of Biology-Ecology and Evolution, University of Fribourg, Fribourg, Switzerland

^6^School of Natural Resources and Environment, University of Florida, Gainesville, FL, USA

^7^Département de Biologie, Faculté des Sciences, Université de Sherbrooke, Sherbrooke, Quebec, Canada

^8^Department of Biology and Marine Biology and Maine Agricultural and Forest Experiment Station, University of North Carolina-Wilmington, North Carolina, USA

^9^Department of Wildlife, Fisheries, and Conservation Biology, University of Maine, 04469 ME, USA

**Contact and corresponding author:**

*Benjamin Baiser [bbaiser@ufl.edu](mailto:bbaiser@ufl.edu)

**Appendix S1:** Sampling site information

**Table S1.** Detailed information for the sites sampled across North America in 2022. Site abbreviation codes are referenced in Figure 1. The Sites are organized by increasing latitude. The temperature data was acquired from two HOBO data loggers that were placed at each field site. These data loggers recorded temperature (°C) every 60 minutes during the entire sampling period (designated as ‘Days’ in the table). The ‘Max Temp’ and ‘Min Temp’ are the highest and lowest recorded temperature values at each field site, respectively. The ‘Mean Temp’ is the average temperature from all recorded temperature data at each field site.

| **Site** | **Location** | **Name** | **Latitude** | **Longitude** | **Sampling Dates** | **Days** | **Max Temp (°C)** | **Mean Temp (°C)** | **Min Temp (°C)** | **Habitat** |
| --- | --- | --- | --- | --- | --- | --- | --- | --- | --- | --- |
| FL | Florida, USA | Crystal Bog | 30.19 | -84.90 | May 12 – August 4 | 84 | 51.7 | 30.4 | 21.2 | Wet Savanna |
| GA | Georgia, USA | Tattnall | 32.16 | -81.99 | May 13 – August 8 | 87 | 48.1 | 28.7 | 19.1 | Wet Savanna |
| NC | North Carolina, USA | Green Swamp Preserve | 34.09 | -78.29 | July 6 – October 15 | 100 | 47.1 | 25.8 | 12.1 | Wet Savanna |
| IN | Indiana, USA | Pinhook Bog | 41.62 | -86.85 | June 7 – August 30 | 84 | 44.1 | 22.1 | 10.8 | Quaking/ Ombrotrophic Bog |
| MA | Massachusetts, USA | Harvard Forest | 42.53 | -72.18 | June 2 – August 8 | 67 | 55.3 | 26.5 | 13.1 | Ombrotrophic Bog |
| WI | Wisconsin, USA | Cedarburg Bog | 43.38 | -88.00 | June 7 – August 31 | 85 | 49.6 | 20.4 | 7.6 | Fen |
| QC | Québec, Canada | Johnville | 45.34 | -71.74 | June 20 – Sept 12 | 84 | 45.0 | 20.5 | 5.0 | Ombrotrophic Bog |

**Appendix S2:** Extended DNA extraction, sequencing, and bioinformatics methods

***DNA Extraction and Sequencing***

We selected 10 pitchers from each site, based on meta-data relating to pitcher integrity, prey capture and completeness of the sampling time course. DNA was extracted using a magnetic bead NucleoMag DNA microbiome kit (Macherey-Nagel GmbH Düren, Germany) with a protocol optimized for RNA/DNA Shield preserved samples and semi-automated processing on the Kingfisher Flex system (ThermoFisher Scientific). We thawed sample tubes on ice before adding 400 μL of lysis buffer (Macherey-Nagel, MI1 buffer) and transferred samples to bead-beating tubes pre-filled with 100 μm zirconium beads (Ops Diagnostics, Lebanon, NY. PFMB #100-100-12). We homogenized samples by bead beating for 2 minutes before returning samples to ice. We centrifuged tubes at 13,000 rpm for 2 minute to pellet beads, transferred 500 μL of the supernatant to a deep-well plate with binding solutions before automated extraction (Nucleomag Microbiome with heat Flex program). We then eluted samples into ~50 μL nuclease-free water and stored them at -70°C.

We tested DNA concentrations of the eluted extracts using an Accuclear Ultra High sensitivity dsDNA quantification kit with standards (Biotium #31028). DNA extracts were then shipped to the Argonne National Laboratory environmental sample processing and sequencing facility for amplicon library preparation and sequencing. We identified bacteria using Earth Microbiome Project primers targeting the 16S rRNA gene (515f-926r) and subsequent sequencing on an Illumina MiSeq using 2 x 250 chemistry (Quince et al. 2011; Parada et al. 2016).

***Bioinformatics***

We processed raw sequence data in QIIME2 v.2023.5 (Bolyen et al. 2019) on the University of Wisconsin-Milwaukee High Processing Computing (HPC), Mortimer. To demultiplex sequences, we used the Earth Microbiome Project QIIME2 demux emp-paired command using no-golay error correction. We used the DADA2 plugin (Callahan et al. 2016) to denoise sequences and generate amplicon sequence variants (ASVs). For taxonomic classification of ASVs, we used the classify-sklearn command against a Naive Bayes classifier trained using SILVA 138 99% OTUs from the 515F/806R region of the 16S rRNA gene. We conducted phylogenetic analysis using the QIIME2 fragment-insertion plugin with the SILVA 128 SEPP reference database to obtain a .nwk file for further analysis in R. Using our negative controls and the “prevalence” method in the decontam package (Davis et al. 2018) in R, we identified 11 ASVs as potential contaminants and removed them from all samples. We quality-filtered data to remove all non-prokaryotic ASVs, and only included observations with at least 10 sequences and samples with at least 1,000 sequences. Lastly, we rarefied samples to a minimum sample depth (1041 reads) using the ‘rrarify’ function in the R package *vegan* (Oksanen et al. 2022). After removing samples that had missing covariates, the resulting matrix included 484 pitcher bacterial samples with 3,323 distinct ASVs.

**Literature cited:**

Bolyen, E., Rideout, J.R., Dillon, M.R. *et al.* Reproducible, interactive, scalable and extensible microbiome data science using QIIME 2. *Nat Biotechnol* **37**, 852–857 (2019).

Callahan, B. J., McMurdie, P. J., Rosen, M. J., Han, A. W., Johnson, A. J. A., & Holmes, S. P. (2016). DADA2: High-resolution sample inference from Illumina amplicon data. *Nature Methods*, *13*(7), 581-583.

Davis, N. M., Proctor, D. M., Holmes, S. P., Relman, D. A., & Callahan, B. J. (2018). Simple statistical identification and removal of contaminant sequences in marker-gene and metagenomics data. *Microbiome*, *6*, 1-14.

Oksanen J, et al.(2022). vegan: Community Ecology Package_. R package version 2.6-4 <https://CRAN.R-project.org/package=vegan>.

Parada, A. E., Needham, D. M., & Fuhrman, J. A. (2016). Every base matters: assessing small subunit rRNA primers for marine microbiomes with mock communities, time series and global field samples. Environmental Microbiology, 18(5), 1403–1414.

Quince, C., Lanzen, A., Davenport, R.J., & Turnbaugh, P.J. (2011) Removing noise from pyrosequenced amplicons. BMC Bioinformatics 12: 38.

**Appendix S3:** GLMM tables for taxonomic (Hill numbers q0, q1 and q2) and phylogenetic diversity.

**Table S1.** Best-fit taxonomic α-diversity GLMM us in q0.

| **Conditional Model** | **Estimate** | **Std. Error** | **z-value** | **p-value** |
| --- | --- | --- | --- | --- |
| Intercept | 35.93 | 1.55 | 23.13 | < 2e-16 |
| *Time* | 12.75 | 0.56 | 22.85 | < 2e-16 |
| *Time^2* | -2.67 | 0.62 | -4.34 | <0.001 |
| *volume* | -1.81 | 0.68 | -2.65 | <0.01 |
| latitude | 2.86 | 1.56 | 1.83 | 0.07 |
| *longitude* | -5.81 | 1.55 | -3.76 | <0.001 |
| *pH* | 2.06 | 0.62 | 3.34 | <0.001 |
| Intact Prey | -0.59 | 0.54 | -1.09 | 0.28 |
| Time:Intact Prey | -1.39 | 0.82 | -1.69 | 0.09 |
| **Dispersion Model** |  |  |  |  |
| Intercept | 2.36 | 0.04 | 67.38 | < 2e-16 |
| *Time* | 0.29 | 0.04 | 7.36 | <0.001 |
| *latitude* | 0.15 | 0.04 | 4.13 | <0.001 |
| *longitude* | -0.33 | 0.04 | -9.17 | < 2e-16 |

**Table S2.** Best-fit phylogenetic α-diversity (SES PD) GLMM.

|  | **Estimate** | **Std. Error** | **z-value** | **p-value** |
| --- | --- | --- | --- | --- |
| Intercept | -1.72 | 0.09 | -18.14 | < 2e-16 |
| *Time* | 0.37 | 0.06 | 6.18 | < 0.001 |
| *pH* | 0.24 | 0.08 | 3.06 | 0.002 |
| *Midge* | 0.26 | 0.07 | 3.47 | < 0.001 |
| longitude | 0.13 | 0.10 | 1.40 | 0.16 |
| **Dispersion Model** |  |  |  |  |
| Intercept | 0.21 | 0.04 | 6.13 | < 0.001 |
| *longitude* | -0.14 | 0.04 | -4.00 | <0.001 |

**Table S3** Best-fit taxonomic α-diversity GLMM using hill number q1.

| **Conditional Model** | **Estimate** | **Std. Error** | **z-value** | **p-value** |
| --- | --- | --- | --- | --- |
| Intercept | 13.51 | 0.70 | 19.25 | < 2e-16 |
| Time | 5.85 | 0.37 | 15.97 | < 2e-16 |
| Volume | -0.86 | 0.30 | -2.82 | 0.005 |
| Intact Prey | -0.34 | 0.33 | -1.06 | 0.29 |
| Longitude | -1.49 | 0.67 | -2.21 | 0.03 |
| pH | 0.50 | 0.33 | 1.52 | 0.13 |
| Time:Intact Prey | -1.48 | 0.50 | -2.95 | 0.003 |
| **Dispersion Model** |  |  |  |  |
| Intercept | 1.86 | 0.04 | 52.81 | < 2e-16 |
| Time | 0.44 | 0.04 | 11.71 | < 2e-16 |
| Volume | -0.15 | 0.04 | -3.81 | <0.001 |
| Longitude | -0.27 | 0.03 | -7.93 | < 2e-14 |

**Table S4** Best-fit taxonomic α-diversity GLMM using hill number q2.

| **Conditional Model** | **Estimate** | **Std. Error** | **z-value** | **p-value** |
| --- | --- | --- | --- | --- |
| Intercept | 8.14 | 0.40 | 20.59 | < 2e-16 |
| Time | 3.36 | 0.26 | 13.17 | < 2e-16 |
| Volume | -0.47 | 0.20 | -2.33 | 0.02 |
| Intact Prey | -0.25 | 0.22 | -1.12 | 0.26 |
| Midge | -0.13 | 0.23 | -0.57 | 0.57 |
| Longitude | -0.65 | 0.37 | -1.79 | 0.07 |
| Time:Intact Prey | -1.14 | 0.36 | -3.145 | 0.002 |
| Time:Midge | 0.55 | 0.29 | 1.90 | 0.06 |
| **Dispersion Model** |  |  |  |  |
| Intercept | 1.47 | 0.04 | 41.38 | < 2e-16 |
| Time | 0.47 | 0.04 | 12.43 | < 2e-16 |
| Volume | -0.16 | 0.04 | -4.22 | < 0.0001 |
| Longitude | -0.21 | 0.03 | -5.90 | < 2e-10 |

**Appendix S4:** Additional results from the Latent Dirichlet Allocation Model

**Figure S1.** Plot of estimated Φ matrix from the Latent Dirichlet Allocation model. The Φ matrix contains estimates of the relative abundance of each ASV in each LDA group. This matrix was used to determine the ten most abundant ASVs.

**Figure S2.** Plot of estimated θ matrix from the Latent Dirichlet Allocation model. The θ matrix contains estimates for the proportion of each LDA group in each sampling unit (i.e., pitcher time-point sample).

**Table S1.** Mean values of posterior coefficient estimates for LDA groups. Bold text indicates that the 95% credible interval did not include zero. Posterior distributions for each coefficient per group are in Figure 5 in the main text.

|  | **Time** | **Latitude** | **pH** | **Longitude** | **Mosquito** |  | **Volume** | **Intact prey** | **Midge** | **Flesh fly** |
| --- | --- | --- | --- | --- | --- | --- | --- | --- | --- | --- |
| Group 1 | **0.77** | **-0.91** | **-0.96** | 0.16 | 0.02 |  | -0.08 | 0.09 | 0.01 | 0.07 |
| Group 2 | 0.06 | **0.7** | **-0.32** | 0.17 | **-0.4** |  | **0.46** | 0.14 | 0.06 | -0.07 |
| Group 3 | **-0.82** | **-1.01** | **-0.49** | **0.29** | 0.05 |  | 0.01 | 0.14 | -0.03 | 0.14 |
| Group 4 | **0.49** | **1.14** | **0.3** | 0.04 | -0.12 |  | 0.21 | -0.14 | 0.13 | 0.14 |
| Group 5 | **-1.94** | **0.45** | **0.64** | **-0.68** | -0.15 |  | **-0.75** | -0.16 | -0.17 | 0.05 |
| Group 6 | **-0.21** | **0.77** | 0.19 | **0.3** | **-0.77** |  | 0.1 | **-0.23** | -0.09 | 0.04 |

**Appendix S5:** Successional trajectories for each ecosystem component.

**Figure S1.** Bivariate plots showing each measured component of the *Sarracenia* *purpurea* microecosystem versus time (days) across all pitchers. Both measures of diversity, ASV richness and SES PD, showed an increasing trend over time. The abundance of midge larvae showed a slight increase in the early weeks of succession then leveled off for the remainder of the trajectory. The abundance of intact prey, which serves as the detrital input to the system, and mosquito larvae increased during the first month of succession and then declined, eventually stabilizing. Flesh fly larvae showed no relationship with time while pH showed a slight decreasing trend and volume showed a slight increasing trend. Focusing on the dynamics of individual pitchers within sites (Appendix S5 Figure S2), we observed patterns similar to those of the entire data set, but the variation among pitchers was more apparent.

**Figure S2:** Bivariate plots showing each measured component of the *Sarracenia* *purpurea* microecosystem versus time (days) for individual pitchers (colors) in each site on the following pages.

.

**Appendix S6:** Phylogenetic trees and Phylogenetic Signal Results

**Fig. S1.** Phylogenetic tree of bacterial ASVs. This phylogenetic tree contains ASVs that meet a probability threshold of 0.01 derived from the LDA model (i.e., ASVs had to have a probability > 0.01 of being in at least one LDA group). Orders are colored along the tip of the phylogeny. The heat map showed the probability of occurrence of each ASV in each LDA group.

**Figure S2 is included as a separate PDF labeled “Appendix_S6_Fig_S2” because it was too large to add to the word document.**

**Figure S2.** Phylogenetic tree of bacterial ASVs. This phylogenetic tree contains ASVs that meet a probability threshold of 0.001 derived from the LDA model (i.e., ASVs had to have a probability > 0.001 of being in at least one LDA group). Orders are colored along the tip of the phylogeny. The heat map showed the probability of occurrence of each ASV in each LDA group.

**Table S1**. Phylogenetic signal of LDA-defined groups. Blomberg’s K was calculated to assess phylogenetic signal within groups 1-6 from the LDA model. Analyses were on datasets subset using probability thresholds of 0.01, 0.001. and 0.0001. Adjusted p-values were calculated using the Benjamini-Hochberg false discovery rate. Groups that show significant phylogenetic signal (adjusted p-value <0.05) are italicized.

| dataset | LDA group | Blomberg’s K | p-value | adjusted p-value |
| --- | --- | --- | --- | --- |
| phi 0.01 | Group 1 | 0.027523 | 0.954 | 0.996 |
|  | Group 2 | 0.06188042 | 0.73 | 0.996 |
|  | Group 3 | 0.05735213 | 0.794 | 0.996 |
|  | Group 4 | 0.16052399 | 0.201 | 0.996 |
|  | Group 5 | 0.05632147 | 0.803 | 0.996 |
|  | Group 6 | 0.09669661 | 0.368 | 0.996 |
| phi 0.001 | Group 1 | 0.01485331 | 0.943 | 0.996 |
|  | Group 2 | 0.0362959 | 0.628 | 0.996 |
|  | Group 3 | 0.02339883 | 0.842 | 0.996 |
|  | Group 4 | 0.04737047 | 0.493 | 0.996 |
|  | Group 5 | 0.01028687 | 0.969 | 0.996 |
|  | Group 6 | 0.02683442 | 0.791 | 0.996 |
| phi 0.0001 | Group 1 | 0.00013834 | 0.996 | 0.996 |
|  | Group 2 | 0.02700823 | 0.523 | 0.996 |
|  | Group 3 | 0.00517936 | 0.934 | 0.996 |
|  | Group 4 | 0.02474536 | 0.414 | 0.996 |
|  | Group 5 | 0.00899385 | 0.814 | 0.996 |
|  | Group 6 | 0.01179793 | 0.805 | 0.996 |

**Table S2.** Phylogenetic signal of LDA-defined groups. Pagel’s lambda was calculated to assess phylogenetic signal within groups 1-6 from the LDA model. Analyses were on datasets subset using probability thresholds of 0.01, 0.001. and 0.0001. Adjusted p-values were calculated using Benjamini-Hochberg false discovery rate. Groups that show significant phylogenetic signal (adjusted p-value <0.05) are italicized.

| dataset | LDA group | Pagel’s lambda | p-value | adjusted p-value |
| --- | --- | --- | --- | --- |
| phi_01 | Group 1 | 7.33E-05 | 1 | 1 |
|  | Group 2 | 7.33E-05 | 1 | 1 |
|  | *Group 3* | *0.6426268* | *6.03E-05* | *0.00036161* |
|  | Group 4 | 0.0144967 | 0.73758919 | 1 |
|  | Group 5 | 7.33E-05 | 1 | 1 |
|  | Group 6 | 0.15604775 | 0.18423202 | 0.33161763 |
| phi_001 | Group 1 | 7.33E-05 | 1 | 1 |
|  | Group 2 | 7.33E-05 | 1 | 1 |
|  | *Group 3* | *0.48250155* | *8.80E-13* | *7.92E-12* |
|  | Group 4 | 0.01854106 | 0.06652652 | 0.13305305 |
|  | *Group 5* | *0.05263545* | *0.01809854* | *0.0465391* |
|  | Group 6 | 0.0692343 | 0.03686388 | 0.08294374 |
| phi_0001 | Group 1 | 7.33E-05 | 1 | 1 |
|  | Group 2 | 5.86E-05 | 1 | 1 |
|  | *Group 3* | *0.21638293* | *8.85E-16* | *1.59E-14* |
|  | *Group 4* | *0.01169628* | *0.00278813* | *0.01003727* |
|  | *Group 5* | *0.02939623* | *0.0001974* | *0.00088832* |
|  | *Group 6* | *0.03594925* | *0.00521576* | *0.01564729* |

**Appendix S7** NMDS of LDA groups.

**Figure S1.** NMDS plot of LDA groups highlights the same temporal and latitudinal patterns identified by the LDA model. The first axis separates groups along latitude, while the second axis reflects their positions along a successional gradient (i.e., early or late). NMDS stress = 0.01.

**Appendix S8** Functional and life history characterization of the ten most abundant ASVs for each LDA group.

**Table S1**: Microbial ASV traits as classified by FAPROTAX and manual annotations of potential functions. Bolded Family names indicate cases when FAPROTAX classifications were derived at the Family level.

| **LDA Group** | **Family** | **Genus** | **FAPROTAX Classification** | **Manual Annotations** | **References** |
| --- | --- | --- | --- | --- | --- |
| Group 1 | Erythrobacteraceae | Novosphingobium | aromatic hydrocarbon degradation | xenobiotic, aromatic compound degradation | Gupta et al., 2009 |
|  | **Chitinophagaceae** | Dinghuibacter | aerobic chemoheterotrophy | aerobic, potentially oligotrophic, N-acetyl-β-glucosaminidase activity | Lv et al., 2016 |
|  | Magnetospirillaceae | Telmatospirillum | nitrogen fixation, fermentation | facultatively microaerophilic, chemoorganotrophic, anoxic, microaerophilic, methanogenic | Sizova et al., 2007 |
|  | **Chitinophagaceae** | Puia | aerobic chemoheterotrophy | facultative aerobe, polysaccharide degradation, limited protein, lipid degradation capabilities | Lv et al., 2017 |
|  | **Sphingobacteriaceae** | Mucilaginibacter | nitrate reduction, aerobic chemoheterotrophy | facultatively aerobic, polysaccharide degradation; non-motile | Pankratov et al., 2007 |
|  | Sporomusaceae | Pelosinus | - | degradation of chlorinated solvents, uranium | Beller et al., 2013 |
|  | Chromobacteriaceae | Paludibacterium | - | facultative aerobe, phospha-tase activity, no beta-glucosidase activity | Kang et al., 2016 |
|  | Bryobacteraceae | Bryobacter | aerobic chemoheterotrophy; chemoheterotrophy; | acid-tolerant, non-motile, aerobic, plant sugar metabolism |  |
|  | Clostridiaceae | Clostridium | ureolysis, reductive acetogenesis | Strict anaerobe, circumneutral pH, capability for hydrolysis , cellulolytic, putrefaction | Eckert et al., 2012 |
|  | Chitinophagaceae | Edaphobaculum | aerobic chemoheterotrophy | non-motile, strictly aerobic, chitinase activity |  |
| Group 2 | **Chitinophagaceae** | - | aerobic chemoheterotrophy | possible chitin degradation |  |
|  | Sphingomonadaceae | Novosphingobium | aromatic hydrocarbon degradation | acidophilic, salt-sensitive | Glaeser et al., 2009 |
|  | Acidobacteriaceae | Terriglobus | aerobic chemoheterotrophy | oligotrophic, aerobic, non-motile, extracellular matrix production, acidicophilic | Männistö et al., 2011 |
|  | Chromobacteriaceae | Chromobacterium | nitrate reduction, chitinolysis | pigmented, motile, associated with *Wyeomyia smithii* | Blackburn et al., 2019 |
|  | **Sphingobacteriaceae** | Mucilaginibacter | nitrate reduction, aerobic chemoheterotrophy | facultative aerobe, polysaccharide degradation, non-motile | Pankratov et al., 2007 |
|  | Rhodanobacteraceae | Dyella | aerobic chemoheterotrophy, dark sulfur oxidation, dark thiosulfate oxidation | plant endophytes; motile |  |
|  | **Sphingobacteriaceae** | Mucilaginibacter | nitrate reduction, aerobic chemoheterotrophy | facultative aerobe, polysaccharide degradation | Pankratov et al., 2007 |
|  | Weeksellaceae | Chryseobacterium | nitrate reduction, fermentation | aerobic, pigmented, antibiotic, chlorine resistant; non-motile | Fox et al., 2015 |
|  | Rhodanobacteraceae | Rhodanobacter | dark thiosulfate oxidation, aerobic chemoheterotrophy | acidophilic; non-motile | Kostka et al., 2012 |
|  | Sphingomonadaceae | Novosphingobium | chemoheterotrophy; aerobic chemoheterotrophy | xenobiotic, aromatic compound degradation |  |
| Group 3 | Chromobacteriaceae | - | human pathogens all | facultative aerobe, phospha-tase activity, no beta-glucosidase activity, often pigmented, motile, associated with *Wyeomyia smithii* |  |
|  | Weeksellaceae | Chryseobacterium | fermentation, aerobic chemoheterotrophy, human gut, ureolysis | Gram neg, aerobic, pigmented, antibiotics, chlorine resistant | Fox et al., 2015 |
|  | **Sphingobacteriaceae** | Mucilaginibacter | aerobic chemoheterotrophy | facultative aerobe, polysacc-haride degradation, non-motile | Pankratov et al., 2007 |
|  | **Sphingobacteriaceae** | Mucilaginibacter | aerobic chemoheterotrophy | facultatively aerobe, polysacc-haride degradation, non-motile | Pankratov et al., 2007 |
|  | **Oxalobacteraceae** | Massilia | nitrogen fixation, xylanolysis, ureolysis | aerobic, motile, copiotrophic | Ofek et al., 2012 |
|  | **Burkholderiaceae** | Burkholderia-Caballeronia-Paraburkholderia | nitrogen fixation, dark thiosulfate oxidation, aerobic chemo-heterotrophy, human pathogens, plant pathogen, aromatic hydrocarbon degradation | potential for N_2_ fixation |  |
|  | Chitinophagaceae | - | aerobic chemoheterotrophy | chitin degradation |  |
|  | Chitinophagaceae | - | aerobic chemoheterotrophy | chitin degradation |  |
|  | Caulobacteraceae | Asticcacaulis | aerobic chemoheterotrophy | aerobic, motile | Zhou et al., 2020 |
|  | Enterobacteriaceae | Enterobacter | fermentation, chemo-heterotrophy; human gut; human associated; mammal gut; animal parasites or symbionts; nitrate reduction | facultative aerobes, motile, nitrate reduction |  |
| Group 4 | Crocinitomicaceae | Fluviicola | aerobic chemoheterotrophy | strict aerobe, non-motile |  |
|  | Sphingomonadaceae |  | aromatic hydrocarbon degradation, aromatic compound degradation | xenobiotic, aromatic compound degradation |  |
|  | Comamonadaceae | - | denitrification, nitrate respiration, dark hydrogen oxidation, dark sulfide oxidation, dark sulfur oxidation, dark thiosulfate oxidation, manganese oxidation, fermentation, aerobic chemoheterotrophy, photoheterotrophy | aerobic, motile |  |
|  | **Chitinophagaceae** | Rurimicrobium | aerobic chemoheterotrophy | aerobic, nonmotile | Dahal et al., 2017 |
|  | **Flavobacteriaceae** | - | chitinolysis, nitrate ammonification, nitrite respiration, xylanolysis, fermentation, aerobic chemoheterotrophy, nitrate respiration, nitrate reduction, fumarate respiration, ureolysis | aerobic, microaerobic, chemoorganotrophic | Jooste, Hugo, 1999 |
|  | Clostridiaceae | Clostridium | ureolysis, reductive acetogenesis | Strict anaerobe, circumneutral pH, capability for hydrolysis, putrefaction |  |
|  | Unclassified |  |  |  |  |
|  | Flavobacteriaceae | Flavobacterium | chitinolysis, nitrate ammonification, nitrite respiration, xylanolysis, fermentation, aerobic chemoheterotrophy, nitrate respiration, nitrate reduction, fumarate respiration, ureolysis | motile |  |
|  | **Chitinophagaceae** | Chitinophagaceae | aerobic chemoheterotrophy | chitinase activity |  |
|  | Schlesneriaceae | Schlesneria | xylanolysis; chemoheterotrophy; aerobic chemoheterotrophy; nitrate respiration; nitrate reduction; nitrate reduction | acidophillic, aerobic, meophilic, diverse polymer degradation, nitrite reduction | Kulichevskaya et al. 2007 |
| Group 5 | Sphingobacteriaceae | Pedobacter | aerobic chemoheterotrophy | endophyte, celluloytic, chitnase activity |  |
|  | **Rhizobiaceae** | Allorhizobium-Neorhizobium-Pararhizobium-Rhizobium group | nitrate respiration, nitrate reduction, aerobic anoxygenic phototrophy, ureolysis, nitrogen fixation | potential for N_2_ fixation |  |
|  | Sphingomodaceae | Sphingomonas | nitrogen fixation, ligninolysis, plant pathogen, aromatic hydrocarbon degradation, aromatic compound degradation, aerobic anoxygenic phototrophy, plastic degradation | strict aerobe, endophyte, oligotrophic, metabolically flexible, biodegradative potential |  |
|  | Oxalobacteraceae | Janthinobacterium | nitrogen fixation, xylanolysis, ureolysis | strict aerobes, motile, oxidase/catalase +, chemoorganotroph, occur in temperate, cold climates | Gillis, Logan 2015 |
|  | Unclassified |  |  |  |  |
|  | **Burkholderiaceae** | Burkholderia-Caballeronia-Paraburkholderia | nitrogen fixation, dark thiosulfate oxidation, aerobic chemo-heterotrophy, human pathogens, plant pathogen, aromatic hydrocarbon degradation | Plant growth promotion | Vio et al., 2020 |
|  | **Oxalobacteraceae** | Massilia | nitrogen fixation, xylanolysis, ureolysis | root-associated, opportunistic pathogen | Ofek et al., 2012 |
|  | **Burkholderiaceae** | Burkholderia-Caballeronia-Paraburkholderia group | nitrogen fixation, dark thiosulfate oxidation, aerobic chemo-heterotrophy, human pathogens, plant pathogen, aromatic hydrocarbon degradation | potential for N fixation |  |
|  | Sphingobacteriaceae | Pedobacter | aerobic chemoheterotrophy | endophyte, celluloytic, chitinase activity |  |
|  | Sphingobacteriaceae | Mucilaginibacter | Xylanosis, aerobic chemoheterotrophy | facultatively aerobic, polysaccharide degradation, non-motile |  |
| Group 6 | Weeksellaceae | Elizabethkingia | aerobic chemoheterotrophy, ureolysis | can associate with *Wyeomyia smithii* | Arellano, Coon 2022 |
|  | Pseudomonadaceae | Pseudomonas | invertebrate parasites, human pathogens, plant pathogen, nitrate respiration, nitrate reduction, plastic degradation | plant associated, phosphatase activity | Patten, Glick 2002; Rodriguez, Varga 1999 |
|  | Sphingobacteriales NS11-12 marine group | NS11-12 | - | Organic matter degradation, marine and freshwater habitats | Meziti et al., 2015; Coclet et al., 2019 Morency et al. 2022 |
|  | **Chitinophagaceae** | Pseudoflavitalea | aerobic chemoheterotrophy | aerobic |  |
|  | Hymenobacteraceae | Hymenobacter | nitrate reduction | aerobic, motile, circumneutral pH, β-galactosidase, α-glucosidase, N-acetyl-β-glucosaminidase activities, potential for hydrolysis | Sheu et al., 2018 |
|  | Flavobacteriaceae | Flavobacterium | chitinolysis, nitrate ammonification, nitrite respiration, xylanolysis, fermentation, aerobic chemoheterotrophy, nitrate respiration, nitrate reduction, fumarate respiration, ureolysis | strict aerobe, mesophilic, non-motile, psychrotolerant, metabolizes diverse C polymers | Huang et al., 2014 |
|  | Flavobacteriaceae | Flavobacterium | chitinolysis, nitrate ammonification, nitrite respiration, xylanolysis, fermentation, aerobic chemoheterotrophy, nitrate respiration, nitrate reduction, fumarate respiration, ureolysis | strict aerobe, mesophilic, non-motile, psychrotolerant, metabolizes diverse C polymers | Huang et al., 2014 |
|  | Flavobacteriaceae | Flavobacterium | chitinolysis, nitrate ammonification, nitrite respiration, xylanolysis, fermentation, aerobic chemoheterotrophy, nitrate respiration, nitrate reduction, fumarate respiration, ureolysis | found in insect guts, chitinase activity, motile. | Huang et al., 2014 |
|  | Flavobacteriaceae | Flavobacterium | chitinolysis, nitrate ammonification, nitrite respiration, xylanolysis, fermentation, aerobic chemoheterotrophy, nitrate respiration, nitrate reduction, fumarate respiration, ureolysis | strict aerobe, mesophilic, non-motile, psychrotolerant, metabolizes diverse C polymers | Huang et al., 2014 |
|  | Unclassified |  |  |  |  |

**Literature cited:**

Arellano, A. A., and K. L. Coon. (2022). Bacterial communities in carnivorous pitcher plants colonize and persist in inquiline mosquitoes. *Animal Microbiome* 4:13.

Beller, H. R., R. Han, U. Karaoz, H. Lim, and E. L. Brodie. 2013. Genomic and Physiological Characterization of the Chromate-Reducing, Aquifer-Derived Firmicute *Pelosinus* sp. Strain HCF1. Applied and Environmental Microbiology 79:63–73.

Blackburn, M. B., R. R. Farrar Jr., M. E. Sparks, D. Kuhar, J. D. Mowery, A. Mitchell, and D. E. Gundersen-Rindal. 2019. *Chromobacterium phragmitis* sp. nov., isolated from estuarine marshes. International Journal of Systematic and Evolutionary Microbiology 69:2681–2686.

Coclet, C., C. Garnier, G. Durrieu, D. Omanović, S. D’Onofrio, C. Le Poupon, J.-U. Mullot, J.-F. Briand, and B. Misson. 2019. Changes in Bacterioplankton Communities Resulting From Direct and Indirect Interactions With Trace Metal Gradients in an Urbanized Marine Coastal Area. Frontiers in Microbiology 10:257.

Dahal, R. H., D. K. Chaudhary, and J. Kim. 2017. Rurimicrobium arvi gen. nov., sp. nov., a member of the family Chitinophagaceae isolated from farmland soil. International Journal of Systematic and Evolutionary Microbiology 67:5235–5243.

Eckert, E. M., M. M. Salcher, T. Posch, B. Eugster, and J. Pernthaler. 2012. Rapid successions affect microbial *N* ‐acetyl‐glucosamine uptake patterns during a lacustrine spring phytoplankton bloom. Environmental Microbiology 14:794–806.

Eichorst, S. A., J. A. Breznak, and T. M. Schmidt. 2007. Isolation and Characterization of Soil Bacteria That Define *Terriglobus* gen. nov., in the Phylum Acidobacteria. Applied and Environmental Microbiology 73:2708–2717.

Fox, J. G. 2015. Laboratory animal medicine. Elsevier.

Gillis, M., and N. A. Logan. 2015. *Janthinobacterium*. Pages 1–12 *in* W. B. Whitman, editor. Bergey’s Manual of Systematics of Archaea and Bacteria. First edition. Wiley.

Glaeser, S. P., P. Kampfer, H.-J. Busse, S. Langer, and J. Glaeser. 2009. *Novosphingobium acidiphilum* sp. nov., an acidophilic salt-sensitive bacterium isolated from the humic acid-rich Lake Grosse Fuchskuhle. International journal of systematic and evolutionary microbiology 59:323–330.

Gupta, S. K., D. Lal, and R. Lal. 2009. *Novosphingobium panipatense* sp. nov. and *Novosphingobium mathurense* sp. nov., from oil-contaminated soil. International journal of systematic and evolutionary microbiology 59:156–161.

Huang, F., Y. Zhang, Y. Zhu, P. Wang, J. Lu, and J. Lv. 2014. *Flavobacterium qiangtangensis* sp. nov., Isolated from Qiangtang Basin in Qinghai-Tibetan Plateau, China. Current Microbiology 69:234–239.

Jooste, P. J., and C. J. Hugo. 1999. The taxonomy, ecology and cultivation of bacterial genera belonging to the family Flavobacteriaceae. International Journal of Food Microbiology 53:81–94.

Kang, H., H. Kim, Y. Joung, K.-J. Kim, and K. Joh. 2016. *Paludibacterium purpuratum* sp. nov., isolated from wetland soil. International Journal of Systematic and Evolutionary Microbiology 66:2711–2716.

Kostka, J. E., S. J. Green, L. Rishishwar, O. Prakash, L. S. Katz, L. Mariño-Ramírez, I. K. Jordan, C. Munk, N. Ivanova, N. Mikhailova, D. B. Watson, S. D. Brown, A. V. Palumbo, and S. C. Brooks. 2012. Genome Sequences for Six Rhodanobacter Strains, Isolated from Soils and the Terrestrial Subsurface, with Variable Denitrification Capabilities. Journal of Bacteriology 194:4461–4462.

Kulichevskaya, I. S., Ivanova, A. O., Belova, S. E., Baulina, O. I., Bodelier, P. L., Rijpstra, W. I. C., ... & Dedysh, S. N. (2007). *Schlesneria paludicola gen. nov., sp. nov*., the first acidophilic member of the order Planctomycetales, from Sphagnum-dominated boreal wetlands. *International Journal of Systematic and Evolutionary Microbiology*, *57*(11), 2680-2687

Lv, Y.-Y., J. Wang, M.-H. Chen, J. You, and L.-H. Qiu. 2016. *Dinghuibacter silviterrae* gen. nov., sp. nov., isolated from forest soil. International Journal of Systematic and Evolutionary Microbiology 66:1785–1791.

Lv, Y., Z. Gao, F. Xia, M. Chen, and L. Qiu. 2017. *Puia dinghuensis* gen. nov., sp. nov., isolated from monsoon evergreen broad-leaved forest soil. International Journal of Systematic and Evolutionary Microbiology 67:4639–4645.

Meziti, A., K. A. Kormas, M. Moustaka-Gouni, and H. Karayanni. 2015. Spatially uniform but temporally variable bacterioplankton in a semi-enclosed coastal area. Systematic and Applied Microbiology 38:358–367.

Morency C., Jacquemot L., Potvin M. Lovejoy C. 2022. A microbial perspective on the local influence of Arctic rivers and estuaries on Hudson Bay (Canada). Elementa 10 (1): 00009. DOI: [10.1525/elementa.2021.00009](https://doi.org/10.1525/elementa.2021.00009)

Ofek, M., Y. Hadar, and D. Minz. 2012. Ecology of Root Colonizing Massilia (Oxalobacteraceae). PLoS ONE 7:e40117.

Pankratov, T. A., B. J. Tindall, W. Liesack, and S. N. Dedysh. 2007. *Mucilaginibacter paludis* gen. nov., sp. nov. and Mucilaginibacter gracilis sp. nov., pectin-, xylan- and laminarin-degrading members of the family Sphingobacteriaceae from acidic Sphagnum peat bog. International Journal of Systematic and Evolutionary Microbiology 57:2349–2354.

Patten, C. L., and B. R. Glick. 2002. Role of *Pseudomonas putida* Indoleacetic Acid in Development of the Host Plant Root System. Applied and Environmental Microbiology 68:3795–3801.

Rodrı́guez, H., and R. Fraga. 1999. Phosphate solubilizing bacteria and their role in plant growth promotion. Biotechnology Advances 17:319–339.

Sheu, S.-Y., T.-Y. Hsieh, S.-W. Kwon, and W.-M. Chen. 2018. *Hymenobacter rivuli* sp. nov., isolated from a freshwater creek. International Journal of Systematic and Evolutionary Microbiology 68:1220–1226.

Sizova, M. V., N. S. Panikov, E. M. Spiridonova, N. V. Slobodova, and T. P. Tourova. 2007. Novel facultative anaerobic acidotolerant *Telmatospirillum siberiense* gen. nov. sp. nov. isolated from mesotrophic fen. Systematic and Applied Microbiology 30:213–220.

Vio, S. A., S. S. García, V. Casajus, J. S. Arango, M. L. Galar, P. R. Bernabeu, and M. F. Luna. 2020. Paraburkholderia. Pages 271–311 Beneficial microbes in agro-ecology. Elsevier.

Zhou, X.-K., Y. Huang, M. Li, X.-F. Zhang, Y.-Q. Wei, S.-C. Qin, T.-K. Zhang, X.-J. Wang, J.-J. Liu, L. Wang, Z.-Y. Liu, and M.-H. Mo. 2020. *Asticcacaulis tiandongensis* sp. nov., a new member of the genus Asticcacaulis, isolated from a cave soil sample. International Journal of Systematic and Evolutionary Microbiology 70:687–692.
